# Feasibility of Real-Time Monitoring for Anemia Using Mobile Application Linked With Point-of-Care Testing Device

**DOI:** 10.7759/cureus.32006

**Published:** 2022-11-29

**Authors:** Kashish Vohra, Gomathi Ramaswamy, Kapil Yadav, Abhishek Jaiswal, Surbhi Gupta, Areeba Khanam

**Affiliations:** 1 Community Medicine, All India Institute of Medical Sciences, New Delhi, New Delhi, IND; 2 Community and Family Medicine, All India Institute of Medical Sciences, Bibinagar, Bibinagar, IND; 3 Community Medicine, All India Institute of Medical Sciences, New Delhi, Delhi, IND

**Keywords:** pregnant women, mobile application, digital hemoglobinometer, hemoglobin, testing, anemia

## Abstract

Background

Anemia testing using digital hemoglobinometers (DH) enabled with real-time data visibility improves delivery outcomes, reduces the manual process of keeping records, and strengthens follow-up. In this study, testing of anemia was done by HealthTrender, an innovative mobile-based cloud-connected application, with HemoCue 301 device (HemoCue AB, Ängelholm, Sweden).

Aim

The objective of the study was to assess the feasibility, acceptability and coverage of anemia testing by the HealthTrender mobile application by the end users - Auxiliary Nursing Midwives (ANMs).

Material and methods

An exploratory mixed-method study was conducted for three months, i.e., from 1st January 2020 to 31st March 2020. The study was done at the antenatal clinics (ANCs) of two Primary Health Centres (PHCs) and their subcentres in the Ballabgarh block of Haryana, India. Qualitative data on the feasibility of the HealthTrender application was collected from 13 ANMs involved in the testing of anemia using in-depth interviews. Quantitative data of 1057 pregnant women attending the ANC clinics was also analysed. For data capturing, a Bluetooth dongle connected with HemoCue 301 transferred the hemoglobin (Hb) values to the HealthTrender mobile application and was uploaded to the cloud, displaying it on a webpage in real time.

Result

Among 1057 pregnant women enrolled, the mean (SD) hemoglobin was 10.9 gm/dl (1.5) and the prevalence of anemia was 47%. ANMs reported that the mobile application was easy to use, and half of them were very satisfied with its speed, easy uploading and quickness in reflecting Hb values on the mobile screen. Challenges related to internet connectivity, loss of Bluetooth dongle connection and less manpower with extra work required were expressed by the ANMs. Completion of records was significantly higher for the mobile application (100%) as compared to manual register-based records (81%).

Conclusion

Web-based application HealthTrender used by ANMs for screening pregnant women for anemia was feasible and had high acceptance. Using digital technology increases the reporting and coverage of hemoglobin testing. Information technology use may be highly beneficial for the serial recording of hemoglobin and subsequent management and follow-ups.

## Introduction

The World Health Organization (WHO) recognizes India as one of the countries where anemia is a serious public health problem with more than 40% of the population being anemic [1]. The recently released National Family Health Survey (NFHS)-5 (2019-2021) reported varying degrees of increase in the severity of anemia in India among children aged 6-59 months (67%), adolescent girls (59%), women of reproductive age group (57%) and pregnant women (52%) compared to the previous NFHS data [2].

Anemia is a serious condition especially in pregnancy as it lowers the hematological reserve for the birth of the child. The current WHO antenatal care (ANC) guidelines recommend full blood count testing as the method for diagnosis of anemia and testing women’s hemoglobin (Hb) status at 12, 26, and 36 weeks of pregnancy. WHO also recommends the use of an on-site hemoglobinometer over hemoglobin colour scale for diagnosing anemia in pregnancy when a full blood count testing facility is not available [3].

Anemia Mukt Bharat (AMB), a recently launched (2018) National Health Strategy in India has laid six interventions to focus on various causes affecting anemia in the country [4]. One of the interventions is to use digital hemoglobinometers (DH) as point-of-care tests (POCT) to estimate Hb status. Identification of anemia, especially in mild and moderate anemia remains undetected through clinical signs [5-7], thus use of DH in case of detection of anemia is more accurate and precise.

There are different methods of Hb estimation that are assessed based on its invasiveness, accuracy, specifications, infrastructure requirements and cost [8]. In rural India, where resources are scarce, these battery-operated DHs are simple to use, sturdy for field use [9], and provide Hb results within a few minutes after one drop of blood sample is taken [10].

Some DHs have a Bluetooth-enabled facility to transfer the Hb values of the individuals from the DH to mobile applications, thereafter to the cloud or dashboard in real time. This digital technology is one of the research areas gaining global interest [11]. It hastens the reporting process and reduces the manual recording of data [12,13]. Digital health technologies have been used in National Health Surveys, diagnosis and control of disease and point-of-care testing using wearable technologies and mobile applications [11,12,14,15]. Increasing penetration of mobile phones (74% of adults globally and India being the second largest user in the world) enables monitoring in facility- and community-based healthcare delivery [16].

Under AMB strategy, a dashboard is designed as a one-stop portal for reporting, monitoring and review mechanism. Details on the prevalence of anemia, distribution of iron folic acid tablets, etc. are entered manually in registers and available on a dashboard [17]. This involves mistakes in documentation, data manipulation, and rewriting, leading to an increase in error and reduced operational efficiency [18]. Similarly, at the primary health care level, frontline workers, such as Auxiliary Nursing Midwives (ANMs), are involved in Hb testing of pregnant women at each ANC visit and documenting it in a register manually.

Thus, there is a need for real-time data capture for anemia management using digital technologies like mobile phones at the peripheral level by healthcare providers. For the detection of anemia, various studies on smartphone-based technologies are available, including HemoGlobe (Johns Hopkins University, Baltimore, USA), Eyenaemia (Stat Innovations, Melbourne, Australia), smartphone dongle, HemaApp (University of Washington, Seattle, USA) and fingernail mobile application [19]. The feasibility of using these technologies for robust screening of anemia among patients has not yet been well documented [20].

This study aimed to assess the feasibility and acceptability of the HealthTrender mobile application (HemoCue AB, Ängelholm, Sweden) by frontline workers for screening of anemia (using WHO anemia classification) [1] among the pregnant women attending ANC at the subcentres and Primary Health Centres (PHC) under Comprehensive Rural Health Services Project (CRHSP), in Ballabgarh, Haryana, India.

The other objectives were, first, to assess the coverage of testing for anemia among pregnant women attending these ANC to get their hemoglobin estimation done. The second was to determine the completeness and consistency of reporting using the HealthTrender application compared to conventional reporting, and the third was to explore the healthcare providers' (user's) perspectives on the HealthTrender application. To the best of our knowledge, there is no published literature exploring the feasibility of real-time data capture for anemia by frontline workers at the grassroots level.

## Materials and methods

Study design and setting

The present exploratory mixed methods study was conducted from 1st January 2020 to 31st March 2020. It was done at the ANCs of the two PHCs of CRHSP, Ballabhgarh, located in Haryana [21]. CRHSP is a sub-district (secondary level care) hospital, and its intensive field practice area (IFPA) caters to 28 villages through 12 sub-centers and two PHCs with a total population of 158,573 residing in 28,389 households.

Each PHC has six sub-centers, and the study was carried out in these two PHCs - Dayalpur and Chainsa - and their sub-centers. At Dayalpur PHC, six sub-centers were included, namely, Machgar, Chandawli, Nawada, Shahpur Kalan, Garkhera, and Dayalpur sub-center. At Chainsa PHC, other six sub-centers, namely, Ladoli, Atali, Naryala, Fatehpur Biloch, Jawan, and Chainsa sub-centers were included.

As a part of routine care, hemoglobin of all pregnant women attending the ANC are tested and recorded in the hospital records. The ANCs are being conducted once a week at all the PHCs and sub-centers. Approximately 50-60 pregnant women attend the ANC at the PHC and 35-40 pregnant women attend the ANC at the sub-centers on any of the ANC clinic days. The information on age, gestational age, education, occupation, and village are maintained in the registers.

Participants and ethics approval

Primary data on the feasibility of the HealthTrender application was captured from 13 ANMs working at the PHC and sub-center level. These ANMs are involved in the estimation of hemoglobin among all pregnant women attending the ANC using digital hemoglobinometers (HemoCue 301). For the study, pregnant women attending an ANC during the study period, irrespective of the trimester and gravida index, were recruited.

The frontline workers who were not involved in the testing of anemia were excluded from the study. Institute Ethics Committee clearance (Ref. No.: IEC-751/04.10.2019) was obtained from the All India Institute of Medical Sciences, New Delhi, India. Participant Information Sheet in Hindi language (local language) was given to each participant (ANM) and pregnant woman, who were enrolled in the study before entering their details into the HealthTrender mobile application.

Description of devices

HemoCue Hb 301 System (HemoCue AB, Ängelholm, Sweden)

It is a battery-operated, pre-calibrated device which consists of a disposable reagent-free microcuvette. This device estimates Hb by measuring the absorbance at two wavelengths (570 nm and 880 nm) in order to compensate for turbidity [22].

HemoCue HealthTrender (HemoCue, Ängelholm, Sweden)

**Figure 1:**
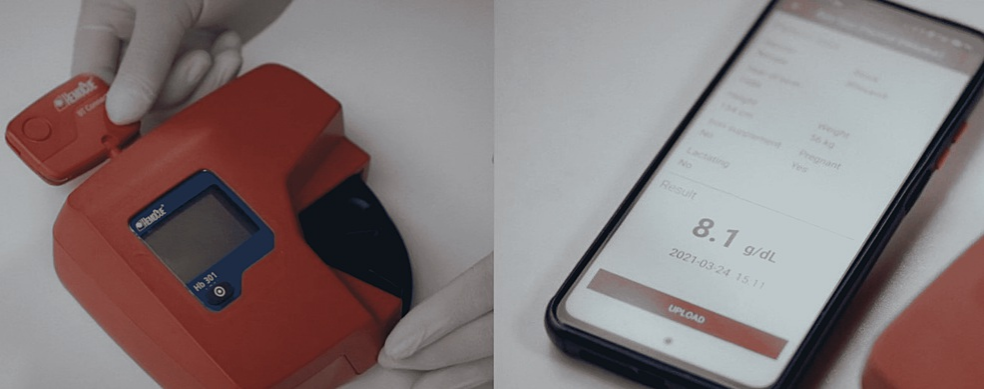
Real-time data transfer from the HemoCue 301 machine to the pre-installed HealthTrender mobile application via a Bluetooth dongle

The HemoCue HealthTrender mobile application is used with HemoCue Hb 301 device equipped with the HemoCue BT Connect Bluetooth dongle. It automatically captures the Hb levels of the subjects from the HemoCue 301 devices thorough a Bluetooth transferring dongle and further transfers it to a smart cloud (Figure 1) [23]. This mobile application can be downloaded from the Google Play store and can be used in Android-based tablets and mobile phones. It gives real-time anemia data in terms of sub-population and geographic region - an immediate insight - thereby eliminating manual entries.

**Figure 2:**
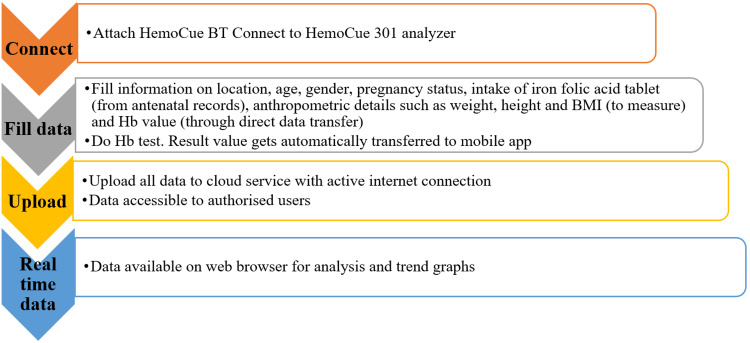
Flow of data from the HealthTrender mobile application to the HealthTrender dashboard. Hb: hemoglobin

Under routine ANC, a second drop of capillary blood samples of the pregnant women was taken for the estimation of Hb. The flow of data transfer is shown in Figure 2. All this data capturing can be done either offline (without an internet connection) and also online (with an internet connection) on mobile phones/tablets. Once the phone has an internet connection, all the data is uploaded to the cloud. In case of no internet connectivity, the data will be stored in the mobile application and uploaded only when the internet network is available. It will take between 5 to 10 seconds for the data to be uploaded to the cloud in presence of internet.

The details of the pregnant women were entered manually into the mobile application by the ANMs, except for the Hb level, which was captured directly. Once the collected data was uploaded to the server, the Hb value and severity of anemia based on gender and predefined age groups display on the HealthTrender webpage. All this data can only be viewed either by investigators of the project or the technical team of HemoCue from the dashboard, and further, the entered data cannot be modified. 

Data collection

Quantitative Data Collection

The feasibility of using the HealthTrender mobile application was captured from the ANMs. For this, the ANMs were given preliminary on-site training by study researchers and manufacturers to accustom them with the functioning of the mobile application technology. After preparing the training protocol and other logistics, a one-day training was carried out at both PHC Dayalpur and PHC Chainsa. ANMs (six to seven) were invited to each of the PHCs and given training on handling the device, with hands-on practice. Also, the data of the pregnant women attending ANC at the sub-centers and at the PHC under CRHSP was extracted from the ANC records and entered in an android-based mobile application tool Epicollect5 (https://five.epicollect.net/).

HemoCue HealthTrender Kit

**Figure 3:**
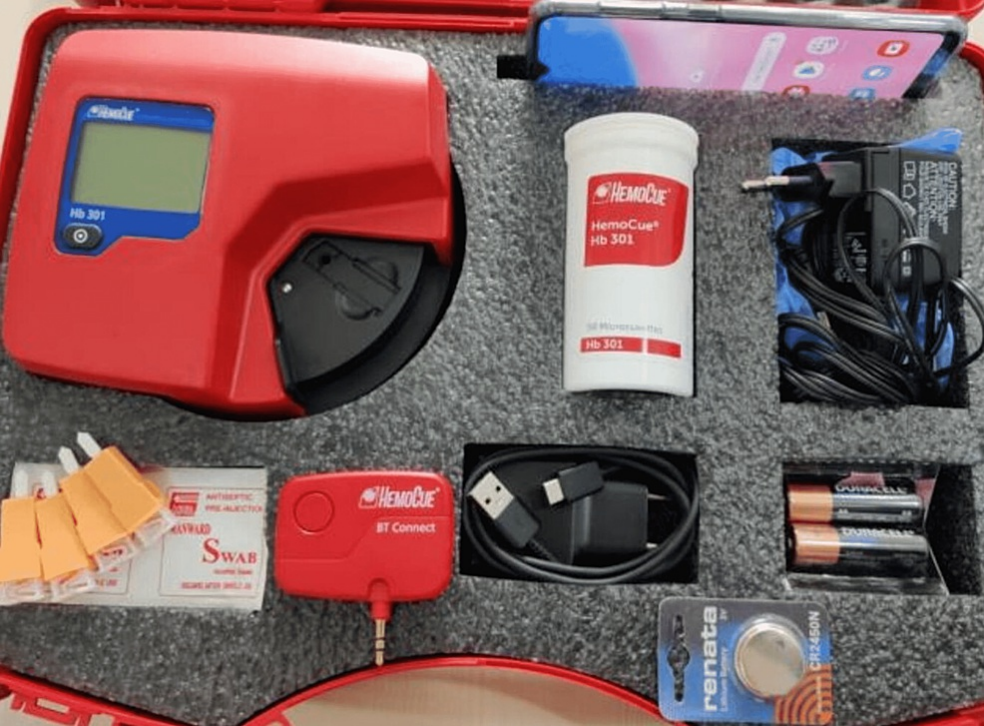
HemoCue Kit containing one mobile device with pre-installed HealthTrender application, one phone charger, one Bluetooth dongle, one HemoCue 301 machine with consumables and battery

Each ANM was given a kit as shown in Figure 3. Total of 13 kits were given, one each for the six sub-centers and one extra for PHC Dayalpur, as two ANC screening rooms were created in one PHC. Customer account IDs were created and given to ANMs to enter data in the mobile application. Authorised users, i.e., investigators of the project, had access to the dashboard to conduct analyses of the data. Investigators contacted the ANMs at the sub-center and at the PHC over the phone to address the issues encountered with HealthTrender. Twice weekly field visits were also made by the investigator and the technical team from HemoCue to monitor the use of HealthTrender and to solve the problems associated with its usage.

Qualitative Data Collection

The end users of the HealthTrender application, i.e., the ANMs, were interviewed at the end of the data collection by an investigator trained in conducting qualitative studies. Semi-structured proforma and in-depth interviews schedule were used by the investigator to explore the feasibility and the challenges encountered with HealthTrender (Table 5 in Appendices). The responses of the ANMs to each question asked were documented in terms of preference on a scale of 'very much' preferred to 'not at all' preferred (5-point Likert scale).

Data management and analysis

The quantitative data (data from antenatal records and data from healthcare worker interviews) was entered manually into the EpiCollect5 mobile application and analyzed with the help of EpiData Analysis V.1.4 (The EpiData Association, Odense, Denmark) statistical software. The results of consistency and completeness of the Hb levels entered in the HealthTrender application compared to the antenatal records were expressed in percentages. The data on the feasibility of the HealthTrender app was also expressed as percentages. The proforma and in-depth interviews were in the Hindi language, and then the transcripts were translated into English. To check the accuracy, summative content analysis was done to explore the challenges and ease of use of HealthTrender.

The data were entered in Microsoft Excel version 2019 (Microsoft Corporation, Redmond, USA) and analyzed using Stata 12 software (Stata Corp, College Station, USA). The continuous variables such as age, hemoglobin values, etc., were summarized using mean (S.D.) or median (IQR) based on the distribution of the data. The categorical variables, such as status of anemia, intake of iron and folic acid tablet and response of the participants, were summarized using percentages. Deductive manual content analysis was done using a set of codes and categories. Quotes of the participants expressed during the in-depth interviews were reported.

## Results

In total, 1057 pregnant women attending ANCs during the study period were enrolled. Out of these, 38.5% (n= 407) were from the Dayalpur PHC and its sub-centers and 61.5% (n=650) were from the Chainsa PHC and its associated sub-centers. The mean (SD) age of the pregnant women were 24.4 (3.7) years. Majority of the them were in the age group of 21-25 years (56.6%). Tables 1, 2 show the demographic, anthropometric, and biochemical values of the pregnant women tested for anemia. Mean (SD) Hb of the pregnant women were reported as 10.9 (1.5) gm/dl. 47% of the pregnant women were found out to be anemic. In terms of severity of anemia, 24.9% (n=263) of the women were mildly anemic, 20.7% (n=219) of them were moderately anemic, and approximately 1.4% (n=15) had severe anemia [1]. One-fourth of the women (23.2%) reported that they were not taking iron folic acid (IFA) tablets.

**Table 1:** Characteristics of the pregnant women attending antenatal clinics at the sub-centers and Primary Health Centres of Dayalpur and Chainsa, Haryana, India

Characteristics		Variable	Frequency (%)
Place		Chainsa	650 (61.5)
		Dayalpur	407 (38.5)
		Total	1057 (100)
Age groups (n=1057)		<=20	129 (12.2)
		21-25	598 (56.6)
		26-30	275 (26)
		31-35	40 (3.8)
		36-40	13 (1.2)
		>40	2 (0.2)
Classification of BMI in Kg/m^2 ^ (n=1002)		<18.5	129 (12.9)
		18.5-24.9	624 (62.3)
		25.0-29.9	197 (19.6)
		>=30	52 (5.2)
Classification of Hemoglobin in g/dl (n=1057)		>=11.0	560 (53)
		10.0-10.9	263 (24.9)
		7.0-9.9	219 (20.7)
		<7.0	15 (1.4)
Iron and folic acid supplements intake (n=1057)		Yes	812 (76.8)
		No	245 (23.2)

**Table 2:** Anthropometry and hemoglobin estimation of the pregnant women attending antenatal clinics at the sub-centers and Primary Health Centres of Dayalpur and Chainsa, Haryana, India

Variable	Mean (S.D.)
Age (in years) (n=1057)	24.4 (3.7)
Height (cm) (n=1013)	154.4 (6.4)
Weight (Kg) (n=1005)	54.5 (11.6)
BMI (in Kg/m^2^) (n=1002)	22.7 (3.9)
Hemoglobin in g/dl (n=1057)	10.9 (1.5)

From Dayalpur’s sub-centers, the highest prevalence of anemia was reported from the Nawada subcentre (53.9%) and Dayalpur PHC (51.1%). From Chainsa, three sub-centers reported the highest prevalence of anemia, namely, Ladoli (57.5%), Fatepur Biloch (52.8%) and Jawa (51.2%). The mean hemoglobin values reported from HealthTrender were also depicted across all the study sites. The highest mean Hb was reported in Atali (11.2 g/dl) and lowest in Naryala subcentre (10.1 g/dl) (Table 3).

**Table 3:** Proportion of anemia and mean (SD) of hemoglobin among pregnant women attending antenatal clinics across different study sites of Primary Health Centers and its sub-centers, Haryana, India PHC: primary health center

S.No.	Sites	Total Sample (n)	Mean Hemoglobin g/dl (SD)	Anemia n (%)	Severity of Anemia		
					Mild Anemia n (%)	Moderate Anemia n (%)	Severe Anemia n (%)
1	Dayalpur sub-center	26	11 (1.1)	12 (46.1)	10 (38.4)	2 (7.6)	0 (0.0)
2	Garkhera	56	11.2 (1.2)	18 (32.1)	9 (16.0)	9 (16.0)	0 (0.0)
3	Machgar	31	10.9 (1.2)	12 (38.7)	6 (19.3)	6 (19.3)	0 (0.0)
4	Chandawli	114	11 (1.3)	51 (44.7)	29 (25.4)	22 (19.3)	0 (0.0)
5	Nawada	113	10.8 (1.7)	61 (53.9)	34 (30.0)	23 (20.3)	4 (3.5)
6	Shahpur Kalan	22	10.3 (3.4)	7 (31.8)	5 (22.7)	1 (4.5)	1 (4.5)
7	Dayalpur PHC	45	10.4 (2)	23 (51.1)	15 (33.3)	8 (17.7)	0 (0.0)
8	Chainsa PHC	295	11.1 (1.6)	132 (44.7)	64 (21.6)	64 (21.6)	4 (1.3)
9	Naryala	20	10.1 (1.6)	6 (30.0)	4 (20.0)	2 (10.0)	0 (0.0)
10	Atali	48	11.2 (1.7)	22 (45.8)	11 (22.9)	10 (20.8)	1 (2.0)
11	Ladoli	66	10.5 (1.4)	38 (57.5)	19 (28)	19 (28.7)	0 (0.0)
12	Fatehpur Biloch	104	10.8 (1.4)	55 (52.8)	31 (29.8)	23 (22.1)	1 (0.9)
13	Jawa	117	10.3 (1.7)	60 (51.2)	26 (22.2)	30 (25.6)	4 (3.4)

In order to check the consistency of Hb values between the HealthTrender application and the ANC records by the ANMs, we compared and cross-checked the values recorded. Discrepancies were found between both data sets. Table 4 shows the various characters of both data sets. Complete data recorded from the HealthTrender was more in pregnant women, i.e., 1057 (100%), than it was recorded in the regular ANC register, i.e., 854 (81%).

**Table 4:** Distribution of various characteristics between the HealthTrender application-generated data set and the ANC register recorded during the same time period ANC: antenatal clinic

Characteristics	HealthTrender application	ANC record
Data recorded among pregnant women	1057	854
Age (Mean, S.D.) (in years)	24.4 (3.7)	23.7 (3.2)
Age (Median, IQR) (in years)	24 (22-26)	23 (21-26)
Age (Range) (in years)	18-49	18-36
Hemoglobin (Mean, S.D.) (in gm/dl)	10.9 (1.5)	10.9 (1.3)
Hemoglobin (Median, IQR) (in gm/dl)	11.1 (10.1-11.9)	11 (10.1-11.9)
Hemoglobin (Range) (in gm/dl)	3.8-15.2	5.9-15.2
No Anemia (%)	53.0	51.5
Mild Anemia (%)	24.9	28.1
Moderate Anemia (%)	20.7	19.3
Severe Anemia (%)	1.4	1.1

**Figure 4:**
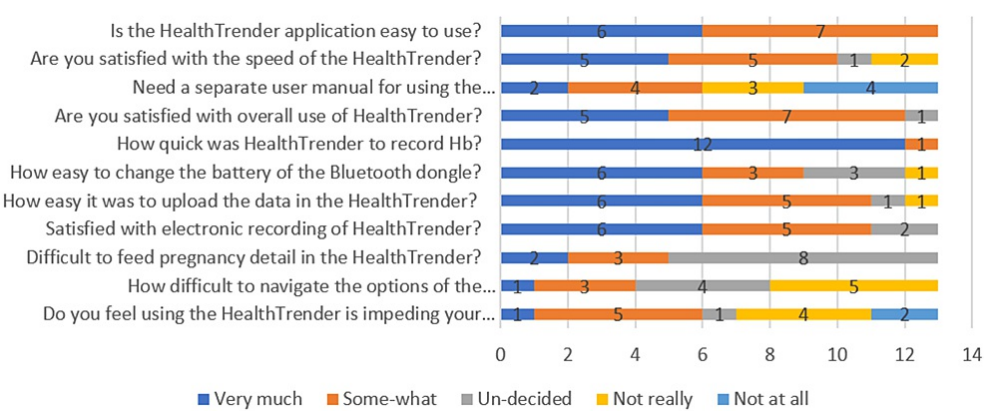
Responses to the feasibility questionnaire from the ANMs on using HealthTrender mobile application among pregnant women at antenatal clinics, India Hb: hemoglobin; ANM: Auxiliary Nursing Midwife

Apart from capturing the coverage in Hb estimation among the pregnant women, 13 ANMs were asked about their experiences while handling HealthTrender through a semi-structured questionnaire and in-depth interview (Figure 4). The majority of the ANMs felt that the HealthTrender mobile application was somewhat easy to use, and half of them were very much satisfied with its speed but felt that a separate user manual is required while using the application.

All ANMs were very much satisfied with the mode of electronic recording, easy uploading and navigating through the options and quick in reflecting Hb values on the mobile screen. Few considered it as an additional work of recording through a mobile application and writing the same in the register. The process of real-time monitoring of anemia was welcomed by the ANMs and felt it to be replicated on other sets of records too. The major challenge associated with real-time monitoring was a dependency on the availability of internet connectivity. Another challenge was the availability of mobile phone devices equipped with the facility of Bluetooth and/or internet connection required for transferring of data.

**Figure 5:**
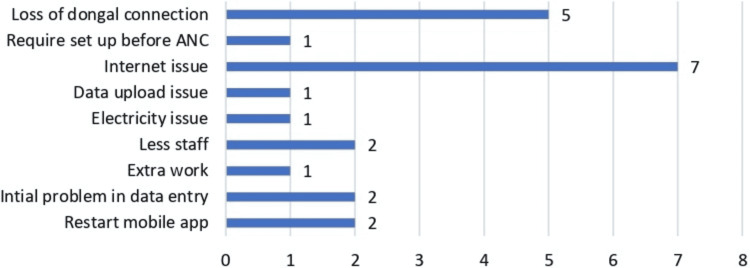
Responses for challenges faced while using the HealthTrender mobile application by ANMs for testing of pregnant women at antenatal clinics, India ANM: Auxiliary Nursing Midwife; ANC: antenatal clinic

When asked regarding the time taken by HealthTrender application in recording one complete entry of pregnant women, the ANMs' responses ranged between 2 and 9 minutes with the mean (S.D.) time taken being 4 minutes 19 seconds. Figure 5 highlights the responses received from ANMs on challenges faced while using HealthTrender. The maximum responses were related to internet connectivity issues. Other feedback included loss of Bluetooth dongle connection, less manpower with extra work required, initial setup and rebooting of the application, and operational setup of application before ANC clinic begins, and, finally, data upload and electricity issues were among the few reasons listed.

In-depth interviews were also taken of 13 ANMs on their experience in handling the HealthTrender, which ranged from 10 to 15 minutes each. One of the responses from the ANMs regarding the usage of HealthTrender was, “The overall process was easy and not difficult for me as we have been trained during the training session.” Another one said, “It was easy and provides results fast.” A few challenges expressed by the ANMs were, “The main problem was of internet connectivity as we were getting trouble in reconnecting,” and “Sometimes it lost connection with the dongle in between a test, so it becomes hectic for us.” 

Most found that the application has an easy-to-use interface with no time-related challenge and was as good as the digital haemoglobinometer they had used previously. Some of the other challenges expressed were: “Once the HemoCue device’s consumables finish, we have to wait for the next available stock;” “We could not retrieve or cross-check the details of patients filled by us;” and “Our workload increased due to double entry in the register and in the mobile application.” A few of the pregnant women didn’t have their date of birth mentioned on their ANC card or forgot, due to which it took more time for the ANMs to do the respective entry in the mobile application.

## Discussion

This study from India provides estimates by the frontline workers, i.e., ANMs on the acceptance and feasibility of real-time monitoring for anemia using the HealthTrender mobile application linked with the HemoCue 301 device. ANMs are currently inundated by a plethora of activities related to national-level programs and keeping manual entries of records. This application cum real-time mechanism generates multiple data sources in enabling frontline workers to capture data timely, researchers and policymakers to make informed decisions, and doctors to improve treatment procedures and detect anemia well in advance.

ANMs tested the Hb levels of pregnant women attending ANCs at two PHC centers, i.e., Dayalpur and Chainsa and at their sub-centers under CRHSP, Ballabhgarh, Haryana, India. In total, 1057 pregnant women were enrolled and 47% of pregnant women were found to be anemic. As observed elsewhere, in the field setup, feasibility and acceptability of real-time monitoring are associated with technological and operational barriers. Interruption in communication networks, user acceptance, inconsistency of interoperability between existing records with electronic health records and system costs involved for implementation are some of the challenges [13].

In this study, the ANMs were asked about the barriers and enablers for the uptake of the HealthTrender mobile application. Positive responses were captured around its smooth interface, satisfactory speed, and electronic data recording. However, a few challenges were also highlighted by ANMs related to data upload, internet and electricity connectivity, initial operational setup and in between loss of Bluetooth dongle connection.

Studies from low- and middle-income countries (LMICs) showed that the mode of record keeping through mobile applications was perceived to be superior to manual records due to its operability, satisfaction and transparency [24]. Thus, a comparison between the manual and digital mode of record keeping by ANMs was done and data entries of Hb values and other ANC records were checked.

The number of entries captured through the mobile application was found to be more, i.e., 1057 (100%), as compared to routine ANC manual register entries which were 854 (81%) in number. The consistency of data differs due to many reasons. Firstly, there was no common identifier in the entries of the HealthTrender application and manual register entries, therefore, it was difficult to match the data on a one-to-one basis. Moreover, in case of the absence of internet or electricity, data entries were firstly stored in the mobile application and later uploaded to the cloud once connected to the network/power. This leads to entering data both manually and uploading online at different times.

The second reason could be errors in the manual entry of data or forgetting to enter a specific record as the manual entry takes twice the time to enter the data than digital entries. Hence it is important to tap on alternate channels by switching to the electronic mode of record keeping in order to avoid loss of information. 

Before the commencement of data collection quality, training was given to frontline workers with on-site monitoring and hand-holding, which increased the validity of the results. The strength of the study is that it was conducted in a field setup which gives an important insight into the actual usage of the device at the field level. The strength of the device included early detection, monitoring and diagnosis of anemia in the study population. Through this mobile application, real-time monitoring of anemia prevalence will be captured at a macro level. It also helps in the incorporation of the digital mode of record keeping and empowers frontline workers to monitor the hemoglobin levels instantaneously and remotely.

It can be inferred from the study that frontline workers will be able to detect anemia through a Bluetooth-enabled device by using a smartphone. There were certain limitations with the HealthTrender device as well as the overall study conducted. With HealthTrender mobile application, a repeat visit by the patient cannot be differentiated from a new visit due to the absence of a unique identifier of the patient. Because of this reason, it will be difficult to provide personalised treatment and care to the patient in follow-up visits as the previous history of Hb level is not interlinked.

One of the limitations of the study was that it captured data only for pregnant women which cannot be compared with the health status of the general population. Record keeping varied across manual data entry of pregnant women when compared to electronic records, thus highlighting human error. The time taken to record a single entry in HealthTrender mobile application was captured but the same was not done for the manual register entries. The study was conducted among frontline workers who were first-hand users of the mobile application with internet and electricity constraints. These results may change if accessed by other professionals. Another limitation includes missing of questions asked to ANMs on recommendation and preference of data capture from the HealthTrender mobile application over manual entries.

## Conclusions

For remote diagnosis, progress in the detection of anemia by using smartphone-based healthcare linked with point-of-care testing devices has been snail-paced. In our study, after using the HealthTrender mobile application, ANMs felt the process to be smooth, easy and quick to record electronic data. One of the major challenges faced was poor internet connectivity. The mean time taken by electronic recording was under 5 minutes. Hence our finding shows important implications for addressing mobile health in the Indian context for testing anemia. Further research is recommended at the grassroots level to reduce misdiagnosis of anemia, a simpler training procedure, and a standard way of measuring observer performance.

## Appendices

**Table 5:** Study questionnaire and in-depth interview on using the HealthTrender mobile application by ANMs for testing of pregnant women at antenatal clinics, India ANM: Auxiliiary Nursing Midwife

S.No.	Question	Very much	Somewhat	Undecided	Not really	Not at all
1	Is the HealthTrender application easy to use?					
2	Are you satisfied with the speed of the HealthTrender application?					
3	Do you need a separate user manual for using the HealthTrender application?					
4	Are you satisfied with electronic recording using HealthTrender application?					
5	How difficult it was to navigate through the options of the Health Trender?					
6	How difficult it was for you to enter the pregnant women details in the HealthTrender?					
7	How easy it was for you to upload the data in the HealthTrender?					
8	How easy it was to change the battery of the Bluetooth dongle?					
9	How quick was the HealthTrender application to record the hemoglobin values?					
10	Are you satisfied with the overall use of the HealthTrender?					
11	Do you feel using the HealthTrender is impeding your regular work?					
12	On an average how much time did it take for to complete the entry of one pregnant woman’s data in HealthTrender? (in minutes)					
13	Did you faced any challenges while using HealthTrender?					
	Guide for In-depth interview					
1.	Ease of using the HealthTrender					
2.	Understanding the interface and navigation through HealthTrender					
3.	Challenges faced while using the HealthTrender					
4.	Time related challenges					
5.	Internet related challenges					
6.	Whether use of HealthTrender affected the routine work?					
7.	Pregnant women related challenges/issues while capturing data in the mobile application?					
